# Patient preferences for stroke prevention treatments in atrial fibrillation in Asia: A discrete choice experiment

**DOI:** 10.1016/j.pmedr.2025.103084

**Published:** 2025-04-25

**Authors:** Rosa Wang, Hui Lu, Gabriela Fernandez, Nicolas Krucien, Dong Huang, Hiroshi Higashiyama, Juan Du, Xin Ye, Tommi Tervonen, Matthew Quaife

**Affiliations:** a Daiichi Sankyo, Inc., Basking Ridge, NJ, United States; b Evidera, London, United Kingdom; c Evidera, Bethesda, MD, United States; d Heart Center, Shanghai Jiaotong University Affiliated Sixth People's Hospital, Shanghai Jiaotong University School of Medicine, Shanghai, China; e Daiichi Sankyo Holdings Co., Ltd, Shanghai, China; f Kielo Research, Zug, Switzerland

**Keywords:** Atrial fibrillation, Stroke prevention, Direct oral anticoagulant, Discrete choice experiment, Treatment attributes, Patient preferences

## Abstract

**Objective:**

Stroke prevention in patients with atrial fibrillation should be adapted to patient needs and preferences. This study quantifies patient preferences for key characteristics of atrial fibrillation stroke prevention treatments in China, Taiwan, and South Korea.

**Methods:**

A discrete choice experiment (DCE) survey was developed based on a targeted literature review and discussions with clinical and methodological experts. The DCE included six attributes: risks of death, severe disabilities, mild or moderate disabilities, non-disabling events; intake with food; and dosing frequency. DCE data were analyzed using mixed multinomial logit models.

**Results:**

In January to March 2023, 307 participants completed the DCE in China (*n* = 155), Taiwan (*n* = 76), and South Korea (n = 76). Average time since atrial fibrillation diagnosis was 6.3 years. Participants preferred treatments with improved clinical outcomes, with reduced risk of death being their most important attribute. They valued a 1 % reduction in severe disability risk the same as a 0.36 % death risk reduction, a 1 % reduction in mild or moderate disability risk the same as a 0.25 % death risk reduction, and a 1 % reduction in non-disabling event risk the same as a 0.18 % death risk reduction. Participant preferences on intake with food and dosing frequency were more heterogeneous than for clinical outcomes.

**Conclusion:**

Patients with atrial fibrillation were willing to accept an increased risk of non-disabling events in exchange for a reduced risk of death but had diverse preferences for treatment administration characteristics. These findings can inform patient-centered treatment strategies that consider the relative importance of treatment attributes in clinical decision-making.

## Highlights

• We studied preferences of patients with atrial fibrillation in Asia.
• Patients preferred stroke prevention treatments with improved clinical outcomes.
• They deemed reduced risk of death to be the most important treatment attribute.
• They would trade off non-disabling event risk for reduced risk of death/disability.

## 1 Introduction

Atrial fibrillation is the most common cardiac rhythm disorder, affecting 2–4 % of adults globally (Hindricks et al., 2021). In Asia, atrial fibrillation prevalence and health burden are predicted to rise substantially, with 72 million cases and three million atrial fibrillation-related strokes by 2050 (Chiang et al., 2014; Kornej et al., 2020).

Atrial fibrillation increases stroke risk up to five-fold (Brundel et al., 2022; Wang et al., 2018) and is associated with 20–30 % of strokes (Kirchhof et al., 2016). Atrial fibrillation also reduces post-stroke survival and increases risks of death and post-stroke disability (Bizhanov et al., 2023; Lamassa et al., 2001), highlighting the importance of preventing stroke in atrial fibrillation (Brundel et al., 2022).

For decades, stroke prevention in atrial fibrillation was warfarin, which is associated with hemorrhage risk and requires careful monitoring (Brundel et al., 2022; Chiang et al., 2016; Kirchhof et al., 2016; Rothberg et al., 2005). The treatment landscape evolved with the direct oral anticoagulants (DOACs) such as dabigatran, rivaroxaban, apixaban, and edoxaban (Chiang et al., 2016; Steffel et al., 2021). DOACs present a safer and more effective alternative to warfarin, showing more predictable pharmacokinetics and fewer food-drug/drug-drug interactions, which enables fixed-dosing, and avoids the need for regular monitoring (Chiang et al., 2014; Chiang et al., 2016; Committee et al., 2012). In Asia, where warfarin-derived issues such as monitoring burden, dietary interactions, and variability in international normalized ratio control are common (Chiang et al., 2014; Zhao et al., 2021), DOACs are preferentially recommended for stroke prevention in atrial fibrillation (Chiang et al., 2014; Chiang et al., 2016). Rivaroxaban and edoxaban are frequently prescribed in Asia-Pacific (Yan et al., 2023).

Stroke prevention in atrial fibrillation should consider treatment differences and patient needs (Hinojar et al., 2015). Patient preference research can facilitate shared decision-making between physicians and patients (Bajorek et al., 2007; Borg Xuereb et al., 2012; Brundel et al., 2022; Rienstra et al., 2024; Van Gelder et al., 2024). DOAC selection for stroke prevention in atrial fibrillation requires understanding patient preferences and the benefit/risk trade-offs they are willing to make.

Research on patient preferences for stoke prevention in atrial fibrillation has primarily compared DOACs with warfarin (Alonso-Coello et al., 2015; Devereaux et al., 2001; Ghijben et al., 2014; Najafzadeh et al., 2014). Comparisons between different DOACs and preference data from the Asia-Pacific region are scarce. Only two studies have explored preferences for different DOACs: one focused on treatment administration (Wilke et al., 2019), and the other did not analyze patient preferences (Tervonen et al., 2017). A further study assessed treatment preferences for oral anticoagulants in China without specifically examining DOACs (Zhao et al., 2021).

This study used a discrete choice experiment (DCE) to quantify DOAC treatment preferences among patients with atrial fibrillation in China, Taiwan, and South Korea, as well as the trade-offs they would be willing to make when selecting a treatment.

## 2 Methods

The study followed best-practice recommendations from the International Society for Pharmacoeconomics and Outcomes Research (Reed et al., 2013). The study protocol was approved on June 27, 2022, by the Ethical & Independent Review Services institutional review board (study number: 22112).

### 2.1 Participants

A convenience sample of adults (≥18 years) living in China, Taiwan, or South Korea was recruited by a third-party vendor (Global Perspectives) between January and March 2023 through patient associations, databases, panels, physician referrals and social media. Eligible participants self-reported a physician diagnosis of atrial fibrillation. Patients were excluded if they had a cognitive impairment, acute psychopathology, or insufficient knowledge of Chinese, Taiwanese, or Korean. For cognitive piloting, patients were also excluded if they had a hearing impairment.

Potential participants received an invitation with information about the study and were screened by trained staff for cognitive pilot telephone interviews. For the quantitative pilot and main DCE, potential participants completed an online screening form. Eligible participants reviewed and completed an online informed consent form before participating in the study. The target sample size (*n* = 300) aligned with typical sample sizes of healthcare DCEs (de Bekker-Grob et al., 2015; Marshall et al., 2010; Soekhai et al., 2019).

### 2.2 DCE survey

#### 2.2.1 Attribute and level development

A targeted literature review on patient preferences for stroke prevention in atrial fibrillation (based on a MEDLINE and Embase searched through Ovid in November 2021) identified key patient-relevant treatment attributes. Clinical data and guidelines on DOACs for stroke prevention in atrial fibrillation were reviewed to identify key differentiators in efficacy and safety profiles and inform attribute level ranges for the DCE (Supplementary Table A.1).

DCE attributes were selected by considering insights from the targeted literature review and clinical data review, a workshop with clinical and methodological experts, cognitive interviews and a quantitative pilot survey. Attributes were intended to be patient relevant, cover a wide range of treatment outcomes and differentiate different DOACs.

For the DCE, six attributes were selected. Four attributes were related to patient-experienced outcomes (fatal events, mild or moderate disabling events, severe disabling events, and non-disabling events). This was because, in the consultation with clinical experts during the study design phase, outcomes such as disabling events were found to be more relevant, understandable, and important to patients than descriptions of different types of clinical endpoints.

The range of patient-experienced outcome attribute levels was determined by mapping to the observed probabilities of relevant clinical endpoints for DOACs and warfarin from major randomized clinical trials and indirect comparisons using warfarin as the common reference (Connolly et al., 2009; Giugliano et al., 2013; Granger et al., 2011; Patel et al., 2011). The level ranges of risks of disabling events were informed by the incidence rates of disabling stroke, myocardial infarction and systemic embolism, and major or life-threatening bleeding, such as intracranial hemorrhage. Stroke severity was defined based on the modified Rankin scale (Joint Commission, 2018). The risk of nondisabling events encompassed non-disabling stroke, myocardial infarction, systemic embolism, major bleeding that will not lead to disabilities (e.g., gastrointestinal hemorrhage, or other major bleeding), and non-major bleeding. Treatment attribute levels were expanded to encompass plausible clinical evidence. To avoid showing very small numbers which risked being ignored or not traded by participants, we converted annual event rates of death and disabling events, which were very low at the lower range, to three-year rates, assuming constant incidence over three years. Two non-clinical treatment attributes (intake with food and intake frequency) were also included due to their patient relevance (Wilke et al., 2019; Moia et al., 2013) and to help differentiate between DOACs with different dosing regimens. Out-of-pocket cost was initially included but was subsequently removed based on the feedback from cognitive pilot interviews (see below 2.2.3).

A no-treatment option was included to reflect real-world decision-making, allowing participants to opt out of the hypothetical treatment options. The attribute levels of the no-treatment option were fixed based on data for the placebo arm from clinical trials of stroke prevention in atrial fibrillation. Clinical experts were consulted to refine the list of patient-experienced outcomes, review event definitions, and determine the ranges of rates for each event. Further detail on attributes and levels is provided in Supplementary Methods and Supplementary Table A.1.

#### 2.2.2 DCE experimental design

**Fig. 1:**
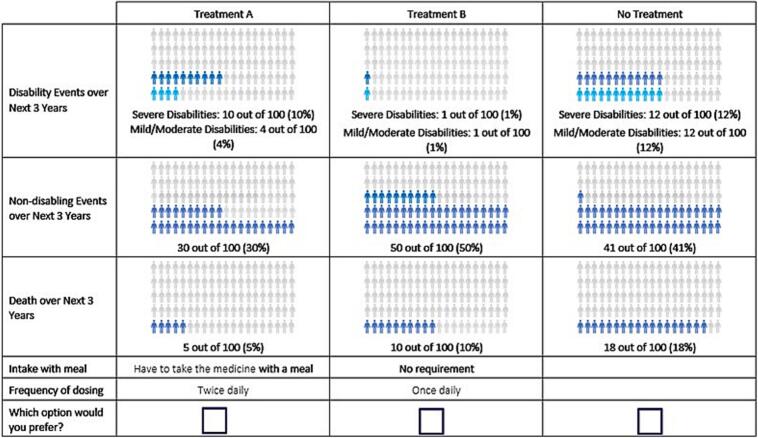
Example of choice task from the discrete choice experiment completed in 2023 by adults with atrial fibrillation in China, Taiwan, and South Korea (*N* = 307). Note: Participants were presented with 12 pairs of hypothetical scenarios and were asked to choose which one they would prefer.

A D-efficient experimental design (Rose and Bliemer, 2009) with 36 experimental DCE tasks was generated with Ngene (version 1.2.1) using directional priors for both the preliminary and final designs. The 36 tasks were split into three blocks with 12 choice tasks each. Each task contained treatment attributes and required participants to choose from two unlabeled hypothetical treatment options and a no-treatment option (Fig. 1). The order of DCE tasks, treatment alternatives and attributes (including groups) were randomized between participants to mitigate ordering, learning, and fatigue bias (Carlsson et al., 2012; Heidenreich et al., 2021).

#### 2.2.3 DCE refinement and finalization

The preliminary survey and DCE design were tested in one-on-one web-based cognitive pilot interviews with 15 participants (five per country). While completing the survey, participants were asked to share their thoughts, which were used to refine the DCE design and improve survey presentation and wording. Additionally, the interviews evaluated whether the DCE attributes and levels were relevant, tradeable, and comprehensible. Participants were also asked if any important aspects were missing. The cognitive pilot interviews reinforced the decision to present outcomes such as disabling events, rather than different types of bleeding, and to use three-year risks to avoid presenting small numbers – the attributes were deemed to be understandable and relevant to participants.

Out-pocket cost was deemed irrelevant to participants in Taiwan and South Korea, where most DOAC costs are covered by health insurance. In China, variable insurance coverage and medicine prices complicated the presentation of realistic cost levels in the DCE. Additionally, cost considerations are complex for patients with comorbidities. Therefore, the cost attribute was removed from the DCE.

To further assess the DCE design, an interim analysis of the refined survey was conducted using data from the first 40 participants. The DCE design was adjusted to avoid presenting the best and worst levels of the death attribute simultaneously in the same choice task, thereby encouraging trade-offs between attributes.

#### 2.2.4 DCE survey flow

Before the DCE, participants were presented with written background information on atrial fibrillation and stroke prevention and were introduced to the concepts of attributes and levels. Participants then completed attribute familiarity tasks, warm-up questions, and a practice task.

During the DCE, participants completed 12 experimental choice tasks. Three non-experimental choice tasks evaluated data quality but were excluded from model analyses. Participants first completed a practice choice task before the experimental tasks, a stability task (task 14 was a repletion of the third task) and a dominance test (Janssen et al., 2017). The dominance test presented a choice between two treatment profiles, one with superior treatment outcomes and identical administration characteristics, to assess survey engagement.

After the DCE, participants completed questionnaires on health literacy and numeracy to assess their ability to understand the choice task information (Supplementary Methods). At survey end, clinical and sociodemographic information was collected.

### 2.3 Data analysis

Descriptive statistics were used to summarize participant characteristics and data on health literacy, numeracy, dominance and stability across tasks, serial non-participation, and response time. To avoid introducing selection bias, no participant was excluded based on failing an internal validity test.

To estimate the impact of treatment attributes on preferences, DCE data were analyzed in a random utility maximization framework (Manski, 1977; Marschak, 1959), which assumes that DCE participants choose the treatment alternative resulting in the highest utility. Different specifications were explored during model development (Supplementary Methods). A fully correlated mixed logit model was estimated for the overall sample to show how participant preferences were influenced by changes in attribute levels (Hensher and Greene, 2003). The model accounted for scale and taste heterogeneity. Marginal utilities (SE and 95 % CI) were used to quantify preferences for key treatment attributes of DOACs.

Marginal rates of substitution were estimated to determine the willingness to swap one attribute change for another providing equal utility; risk of death was chosen as the common metric due to its expected impact on preferences.

Subgroup analyses examined how preferences varied by observable clinical and sociodemographic characteristics, including geographical region, age, education, employment, comorbidities and treatment experience. Preference heterogeneity was explored through interaction effects between attributes and participant characteristics in mixed logit models.

A predicted choice probability analysis was conducted to estimate the proportion of patients expected to choose one treatment over another, given preference model results and clinical performance estimates (Supplementary Methods). Preference and clinical data were combined to understand the expected desirability of specified DOACs and to assess how this desirability depended on their clinical profiles and fatality rates. The estimation captured the uncertainty of the clinical and preference data. 95 % CIs were reported to indicate the precision of the estimates.

All analyses were performed using R version 4.0.5. Unless otherwise noted, all statistical tests were two-sided, used a significance level of 0.05, and were not adjusted for multiple comparisons.

## 3 Results

### 3.1 Demographic and clinical characteristics

Over 3000 individuals were invited to participate and more than 1000 were interested in participating. Of these, 394 were eligible and 307 completed the study; half of them were from China and the other half were equally distributed between Taiwan and South Korea (Table 1).

**Table 1:** Demographic and clinical characteristics of the adults with atrial fibrillation in China, Taiwan, and South Korea (N = 307) who completed the discrete choice experiment.

Characteristic	Overall (N = 307)	China (N = 155)	Taiwan (N = 76)	South Korea (N = 76)
Age (years)				
Mean (SD)	48 (11.6)	50 (11.6)	42 (10.0)	48 (11.1)
Range (min, max)	19–72	19–72	20–61	24–70
Sex (male), n (%)	153 (50)	68 (44)	40 (53)	45 (59)
Employment status, n (%)				
Employed, full-time	227 (74)	90 (58)	71 (93)	66 (87)
Retired	63 (21)	58 (37)	2 (3)	3 (4)
Other⁎	17 (6)	7 (5)	3 (4)	7 (9)
Educational background, n (%)				
High school or less	93 (30)	56 (36)	15 (20)	22 (29)
College or higher	210 (68)	99 (64)	61 (80)	50 (66)
Other	4 (1)	0 (0)	0 (0)	4 (5)
Time since atrial fibrillation diagnosis				
Years, mean (SD)	6.4 (8.2)	6.5 (9.4)	8.2 (8.4)	5.1 (4.2)
Years, median (first quartile, third quartile)	3.8 (2.6–7.1)	3.8 (2.6–5.8)	4.7 (2.7–10.5)	4.0 (2.3–7.2)
Medical history, n (%)				
Cardiovascular disease	114 (37)	53 (34)	27 (36)	34 (45)
Diabetes	49 (16)	21 (14)	18 (24)	10 (13)
Bleeding (minor)	36 (12)	22 (14)	8 (11)	6 (8)
Stroke/transient ischemic attack/systemic embolism	33 (11)	17 (11)	10 (13)	6 (8)
Other vascular disease	29 (9)	5 (3)	18 (24)	6 (8)
Renal impairment	24 (8)	11 (7)	6 (8)	7 (9)
Liver impairment	22 (7)	11 (7)	5 (7)	6 (8)
Cancer	18 (6)	5 (3)	3 (4)	10 (13)
Bleeding (major)	16 (5)	8 (5)	1 (1)	7 (9)
Other thrombosis	16 (5)	7 (5)	3 (4)	6 (8)
No, none of these conditions	113 (37)	68 (44)	12 (16)	33 (43)
Longest time on current anticoagulant treatments, n (%)				
<1 month	6 (2)	1 (1)	3 (5)	2 (3)
1–2 months	17 (6)	6 (5)	6 (9)	5 (7)
3–6 months	44 (17)	20 (15)	7 (11)	17 (25)
>6 months	197 (75)	106 (80)	48 (75)	43 (64)
Not applicable	43 (14)	22 (14)	12 (16)	9 (12)
Current direct oral anticoagulant treatments, n (%)				
Rivaroxaban	132 (46)	87 (53)	21 (33)	24 (40)
Apixaban	75 (26)	39 (24)	18 (28)	18 (30)
Dabigatran	55 (19)	23 (14)	19 (30)	13 (22)
Edoxaban	27 (9)	16 (10)	6 (9)	5 (8)
Previous anticoagulant treatments, n (%)				
Warfarin	54 (18)	17 (11)	23 (30)	14 (18)
Rivaroxaban	90 (29)	56 (36)	19 (25)	15 (20)
Apixaban	51 (17)	26 (17)	7 (9)	18 (24)
Dabigatran	21 (7)	6 (4)	7 (9)	8 (11)
Edoxaban	16 (5)	8 (5)	5 (7)	3 (4)
None of the above	75 (24)	42 (27)	15 (20)	18 (24)
Self-reported overall health, n (%)				
Excellent	45 (15)	14 (9)	19 (25)	12 (16)
Very good	50 (16)	14 (9)	23 (30)	13 (17)
Good	120 (39)	61 (39)	21 (28)	38 (50)
Fair	77 (25)	56 (36)	10 (13)	11 (14)
Poor	15 (5)	10 (6)	3 (4)	2 (3)

⁎ Includes part-time employed, unemployed, student, homemaker/housewife, disabled, other.

The overall participant population covered a wide range of sociodemographic and clinical characteristics (Table 1). The mean age was 48 years (SD 11.6, range 19–72). Women and men were equally represented in the overall sample (50 % each), but the proportion of men was higher in Taiwan (53 %) and South Korea (59 %). Participants were mainly full-time employees (74 %) or retired (21 %) and most had college or higher education (68 %) (Table 1).

The average time since diagnosis was approximately six years (77 months, SD 98.7). Medical histories most frequently involved cardiovascular diseases (37 %), bleeding (12 % minor and 5 % major), diabetes (16 %), stroke (11 %), and other vascular disease (9 %). Most participants (75 %) had received their current anticoagulant treatment for more than six months. Rivaroxaban was the most frequently used DOAC in all three countries (Table 1).

### 3.2 Data validity

Data quality indicators were assessed in line with previous recommendations (Johnson et al., 2019). Most participants had high health literacy, with 76 % scoring more than two points on a five-point scale rating reading problems, and high numeracy, with all scoring three or more points on a five-point numeracy scale (Supplementary Table A.2).

Most participants passed the dominance test (overall 87 %; China 91 %; Taiwan 79 %; South Korea 86 %) and made consistent choices in the stability test (overall 71 %; China 68 %; Taiwan 76 %; South Korea 71 %). Nearly all participants made trade-offs in their decision-making (overall 95 %; China 99 %; Taiwan, 86 %; South Korea: 97 %). Most participants completed the DCE in less than five minutes (overall 86 %; China 84 %; Taiwan 86 %; South Korea 89 %).

### 3.3 Overall sample preferences

**Fig. 2:**
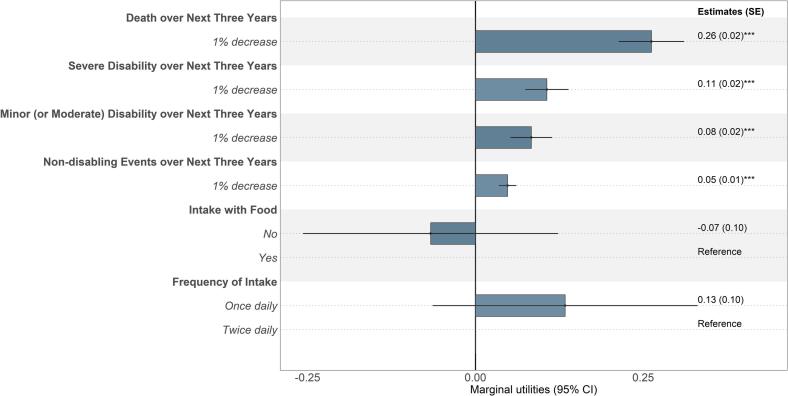
Preference estimates for non-vitamin K antagonist oral anticoagulant therapy in adults with atrial fibrillation in China, Taiwan, and South Korea (N = 307). Note: The mean marginal utility and 95 % CI of each attribute, relative to other attributes included in the DCE, are shown. Estimates are marginal utilities that denote the effect of deviating from a reference attribute level on preferences. Positive mean estimates with a 95 % CI >0 suggest participants place a higher value on the attribute level over the reference level, indicating that participants prefer the treatment option with this attribute level, all else being equal. *** indicates *p* < 0.001. Abbreviations: CI, confidence interval; MLE, maximum likelihood estimates; SE, standard error.

All outcome attributes influenced participants' treatment preferences (*p* < 0.001, Fig. 2). Preferences for attributes with numerical levels were found to be best described by a linear specification. The marginal utility, representing the impact of a change in attribute level on preferences, was inversely correlated to risk reduction of death, severe disabilities, and mild or moderate disabilities (Supplementary Fig. A.2).

Among the four treatment-related outcomes, risk reduction in death over the next three years had the largest impact on treatment preferences, followed by severe disabilities, minor or moderate disabilities, and non-disabling events (*p* < 0.001). Every 1 % risk reduction of death over three years was valued twice as highly as every 1 % risk reduction of severe disabilities and was deemed three times more important than a risk reduction of mild or moderate disability over three years (Fig. 2).

Estimated coefficients for treatment administration attributes (intake with food and frequency of intake) were not statistically significant, indicating greater preference heterogeneity relative to other outcomes (Fig. 2 and Supplementary Fig. A.2).

### 3.4 Treatment attribute trade-offs

**Fig. 3:**
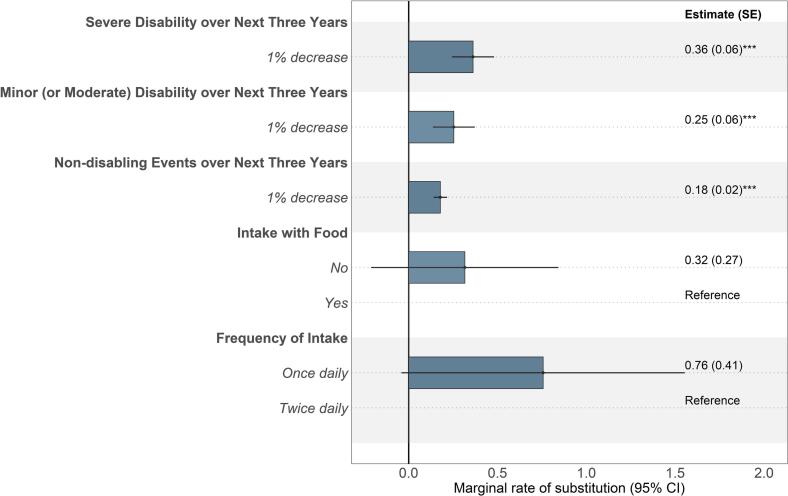
Maximum acceptable risk of death over the next three years in adults with atrial fibrillation in China, Taiwan, and South Korea (N = 307). Note: The minimal improvement in death reduction required by patients to accept 1 % increase in poorer outcomes on other attributes is shown. MRS were estimated to determine the willingness of a patient to swap one attribute change for another providing equal utility. Abbreviations: CI, confidence interval; MLE, maximum likelihood estimates; MRS, marginal rates of substitution; SE, standard error.

While participants were willing to accept increases in certain risks in exchange for improvements in death risk, the trade-off depended on the outcome. Participants required a 0.36 % (95 % CI: [0.24; 0.48]) reduction in death risk to tolerate a 1 % increase in severe disability risk (Fig. 3). Participants required a minimal of 0.25 % (95 % CI: [0.14; 0.37]) or a 0.18 % (95 % CI: [0.14; 0.22]) reduction in death risk over the next three years to tolerate a 1 % increase in risks of minor or moderate disability or non-disabling events. Older participants (≥56 years of age) placed a higher value on death risk reduction over the next three years than younger participants (Supplementary Fig. A.3). Compared to participants without comorbidities, those with comorbidities placed less value on reducing the risks of death and non-disabling events over the next three years (*p* < 0.05; Supplementary Fig. A.4).

### 3.5 Treatment comparison

**Fig. 4:**
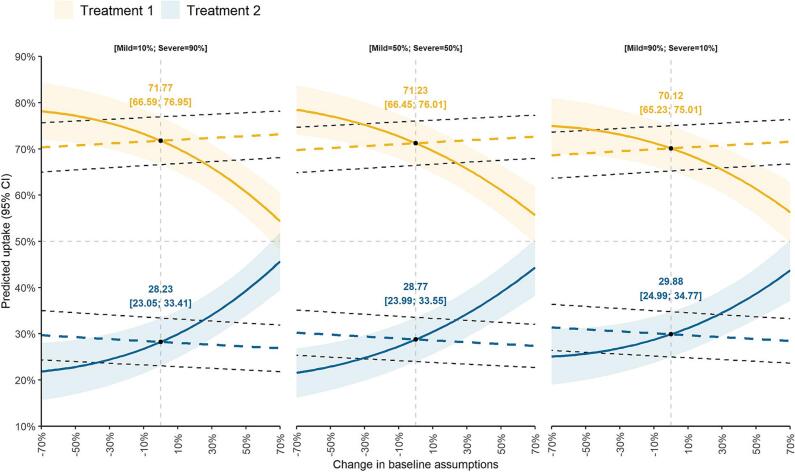
Predicted choice probability analysis of treatment preferences of adults with atrial fibrillation in China, Taiwan, and South Korea (N = 307) under different assumptions of fatal, disabling, and non-disabling event rates. Note: All lines denote the probability of respondents preferring treatment profile 1 (edoxaban-like; solid and dashed yellow lines) over profile 2 (rivaroxaban-like; solid and dashed blue lines) for varying levels of disabling events, non-disabling events, and fatal events (clinical performance is presented in Supplementary Table A.1). The shaded areas and the areas within the black dashed lines indicate 95 % confidence intervals. Each line summarises a change to baseline assumptions in either non-disabling events (solid orange and blue lines) and fatality rates (dashed blue and orange lines) for different ratios of mild and severe disabling events (shown in the three panels), holding all else equal.

On average, participants preferred an edoxaban-like DOAC treatment profile (71.23 %, 95 % CI: [66.45; 76.01]) over a rivaroxaban-like one (28.77 %, 95 % CI: [23.99; 33.55]), over wide parametric ranges (Fig. 4 and Table 2). The probability of the edoxaban-like profile being preferred decreased with higher non-disabling event incidence and increased with greater fatality incidence. Assumptions pertaining to non-disabling event incidence had a larger impact on predicted treatment choice than fatal event assumptions or assumptions of the ratio of mild-to-severe disabling events.

**Table 2:** Clinical performance data for the selected comparison of non-vitamin K antagonist oral anticoagulant therapies.

	Treatment Profile 1 (Edoxaban-like profile)	Treatment Profile 2 (Rivaroxaban-like profile)
Death (any cause)⁎	0.77	0.90
Non-disabling events⁎	12.14	16.22
Mild disabling events⁎	1.55	2.31
Major disabling events⁎	1.77	2.55
Intake with food	No	Yes
Frequency of intake	Once daily	Once daily

⁎ Data are annual incidence rates (%).

## 4 Discussion

### 4.1 Summary of findings

Our results provide insights into the relative importance patients with atrial fibrillation place on key features of DOACs for stroke prevention in China, Taiwan, and South Korea. Reduction in risk of death over the next three years was the most important outcome influencing participant choices, followed by reduction in risk of severe disability, mild or moderate disability, and non-disabling events. Participants were willing to trade an increased risk of non-disabling events for a reduced risk of death or disabilities. Non-clinical attributes (intake frequency and intake with food) influenced treatment choices less than patient-experienced outcomes and were valued heterogeneously by participants from different countries. The predicted choice probability analysis found that participants preferred an edoxaban-like treatment profile over a rivaroxaban-like one.

This study filled a knowledge gap on preferences for DOAC treatment outcomes and administration characteristics among patients with atrial fibrillation in the Asia-Pacific region.

### 4.2 Implications

Prioritization of a lower risk of death and disabilities is consistent with preference literature on stroke prevention in atrial fibrillation. One study reported that patients were willing to accept a 6.3 % (United States) or 2.2 % (Japan) increase in the risk of nonmajor clinically-relevant bleeding for a 1 % reduction in disabling stroke risk (Okumura et al., 2015). Another study found that stroke and bleeding risk were the two most important attributes for 89 % of Australian patients (Ghijben et al., 2014). Additional, a DCE examining patient preferences for anticoagulant therapy revealed that adults with a history of cardiovascular disease would trade a 1 % lower risk of death due to bleeding for a 2.8 % risk of stroke, a 2.2 % risk of myocardial infarction, and a 3.4 % risk of death due to stroke or myocardial infarction (Najafzadeh et al., 2014). These findings suggest that atrial fibrillation patients are willing to trade-off benefits and risks of DOACs, and that clinicians should emphasize these outcomes in discussions with patients to ensure effective shared decision-making for stroke prevention. Our findings on the relatively low importance that participants assigned to food intake align with a previous preference study conducted in China, where food-drug interactions were of least concern for patients unless they had a history of stroke or bleeding (Zhao et al., 2021). These results are also consistent with recent DCE data on DOAC patient preferences among adults with atrial fibrillation in France, the UK, and Germany, where intake with food was the lowest-ranked attribute (10.6 %) (Wilke et al., 2019). These insights into patient preferences can help clinicians in the Asia-Pacific region to personalize treatment such that it aligns with patient preferences.

The out-of-pocket cost attribute was identified as unimportant to patients in Taiwan and South Korea in the quantitative pilot and was therefore removed from the DCE. This is in line with a prior atrial fibrillation stroke prevention treatment preference study from China, which found that the cost attribute was not important for patients with a household income over RMB 5000 per month (58 % of the sample) (Zhao et al., 2021).

### 4.3 Strengths and limitations

Our findings are underpinned by the presentation of outcome attributes as events that can range from non-disabling to disabling or fatal events; in piloting and the main survey, this format was found to be comprehensible and meaningful to patients considering atrial fibrillation treatment options and constitutes a clear strength of the study. A further strength is the large sample achieved via a robust mixed-model multi-phase study design.

To facilitate participant comparisons, we converted the annual event rates to a three-year period, assuming a constant incidence over this timeframe. The results should be interpreted accordingly. Attribute confounding is a general risk within DCEs where attributes are overlapping or the patient preference for one attribute depends upon other attribute levels. Here, DCE design aimed to minimize confounding issues by selecting distinct treatment aspects as attributes. Moreover, participants were explicitly instructed to consider only distinct treatment aspects when making their choice. All stated preference methods, including DCEs, may be subject to hypothetical bias. To mitigate hypothetical bias and make the DCE credible and relevant to patients, our survey design used findings from a targeted literature review and cognitive pilot interviews.

Due to challenges in recruitment in this region, convenience sampling was used. The sample obtained, while broad, may not be generalizable to the larger population of interest. To mitigate potential sampling bias, multiple recruitment channels were used. Additionally, subgroup analysis was conducted to explore the impact of patient characteristics on treatment preferences.

## 5 Conclusions

Patients with atrial fibrillation in China, Taiwan, and South Korea were willing to trade a risk of non-disabling events for a reduced risk of death or disability but had diverse preferences around treatment administration. The present results can inform patient-centered treatment strategies that consider the relative importance of treatment attributes in clinical decision-making. This, in turn, may facilitate shared decision-making between patients and physicians.

## CRediT authorship contribution statement

**Rosa Wang:** Writing – review & editing, Writing – original draft, Visualization, Supervision, Resources, Project administration, Methodology, Investigation, Funding acquisition, Data curation, Conceptualization. **Hui Lu:** Writing – review & editing, Visualization, Supervision, Project administration, Methodology, Investigation, Conceptualization. **Gabriela Fernandez:** Writing – review & editing, Project administration, Investigation, Data curation, Conceptualization. **Nicolas Krucien:** Writing – review & editing, Visualization, Validation, Software, Formal analysis. **Dong Huang:** Writing – review & editing, Supervision, Investigation, Formal analysis, Conceptualization. **Hiroshi Higashiyama:** Writing – review & editing, Supervision, Methodology, Investigation, Conceptualization. **Juan Du:** Writing – review & editing, Supervision, Methodology, Investigation, Conceptualization. **Xin Ye:** Writing – review & editing, Supervision, Methodology, Investigation, Conceptualization. **Tommi Tervonen:** Writing – review & editing, Supervision, Methodology, Investigation, Conceptualization. **Matthew Quaife:** Writing – review & editing, Writing – original draft, Supervision, Investigation.

## Funding sources

This study was funded by Daiichi Sankyo. Inc. The funding did not have any impact on teh study design, data collection, adn analysis, or preparation of the manuscript.

## Declaration of competing interest

RW, HH, JD, and XY are employees of Daiichi Sankyo, Inc., which funded the present study. RW and XY also own stock/stock options in Daiichi Sankyo. HL, GF, NK, and MQ are employees of Evidera, Inc., which received payment from Daiichi Sankyo for work relating to the present study. NK is also a minority stockholder of Thermo Fisher Scientific as part of his employment with Evidera. DH has no disclosures to report. TT is a former employee of Evidera. He is now an employee of, and owns stock in, Kielo Research, which provides patient-centered research services to the pharmaceutical industry, including companies manufacturing atrial fibrillation treatments.

## Data Availability

Data will be made available on request.

## Appendix A Supplementary data

Supplementary material Image 1
