# Supplementary material for: Patient preferences for stroke prevention treatments in atrial fibrillation in Asia: A discrete choice experiment
Source: Prev Med Rep. 2025 Apr 25;54:103084. doi: 10.1016/j.pmedr.2025.103084 (PMC12127864; doi:10.1016/j.pmedr.2025.103084)
Supplement: Supplementary file 1 — Supplementary material [file mmc1.docx]

# Supplementary Appendix a

## 1. Supplementary Methods

### 1.1 Qualitative pilot testing

To ensure the feasibility and robustness of the discrete-choice experiment (DCE) qualitative pilot, cognitive interviews were conducted via telephone with synchronized screen sharing of the DCE materials between the interviewer and participant. The goals of these cognitive pilot interviews were:

- To assess if the chosen DCE attributes and levels were relevant, tradeable, and understandable by participants (Ryan et al., 2009). Two attributes were ‘tradeable’ if participants were willing and able to give up some or all of one attribute to get more (or less) of another attribute.
- To assess the complexity and clarity of the overall questionnaire, including length, number of questions, wording, presentation format, structure, and completeness of response options.

Cognitive pilot interviews were conducted with 15 patients (five from each country). An example DCE choice task was added ahead of the attribute introduction. Survey and attribute introductions were updated for clarity based on pilot participant feedback. Attribute descriptions and examples were adjusted for clarity.

### 1.2 Quantitative pilot testing

The quantitative pilot assessed if the attribute levels covered the preference-relevant range or needed to be updated, identified parameter estimates that could be improved, and gained initial insights into preferences. An interim analysis was conducted to ensure that patients could meaningfully distinguish between different attribute levels, allowing for any final adjustments to the experimental design prior to the main survey launch.

Overall, the quantitative pilot, in combination with the insights from the cognitive interviews aimed to create a survey design that would carefully balance participants’ cognitive burden while maximizing statistical efficiency. The quantitative pilot was conducted among the first 73 participants across the countries of interest to test and refine the design of the preference elicitation instrument. Based on the findings from the first wave of the soft launch, the DCE design was updated so that participants were not presented with choices with best and worst levels of death attribute in the same choice to encourage trade-offs between attributes.

### 1.3 Model development

Different choice models were systematically tested to understand which best fit the DCE data. First, we tested a multinomial logistic regression model with initial dummy-coded specification of all attributes and levels. The multinomial logistic regression model assumed that 1) preferences are homogenous; 2) choices are independent of irrelevant alternatives; and 3) there are no panel effects introduced by the same participant making multiple choices.

Next, we assessed whether the dummy-coded specification of attributes could be simplified by treating continuous/numerical attributes (i.e., outcome attributes with probability levels) as linear variables. For each attribute separately, the assumption of linearity was accepted when the coefficient of determination (R^2^) of a linear regression line through the dummy-coded effects was >80%.

The assumptions of choice independence and preference homogeneity were then simultaneously relaxed with mixed logit models that allowed individual preferences to be distributed within the sample. The mixed logit model relied upon continuous (i.e., normal) distributions of preference weights (Boxall and Adamowicz, 2002; Greene and Hensher, 2003; McFadden and Train, 2000; Revelt and Train, 1998; Swait, 1994). Different specifications of the random effects were compared (e.g., normal vs. lognormal distributions; independent vs. correlated random effects).

Statistical performance was compared across models based on goodness-of-fit statistics, including the Akaike information criterion, Bayesian information criterion, Adjusted McFadden Pseudo-R^2^, and/or the likelihood ratio test.

The final choice model estimated average preferences using a fully correlated mixed logit model that treated all attributes as linear variables, excepting intake with food and intake frequency.

### 1.4 Predicted choice probability

The predicted choice probability calculation involved applying model preference estimates for each attribute to the relevant level of benefit or risk as well as to non-clinical treatment characteristics associated with each treatment (intake frequency and intake with food), using a logit model:

| $P_{j}\boldsymbol{=}\frac{\exp[v\left( \boldsymbol{x}_{j}\boldsymbol{;}\hat{\boldsymbol{\beta}} \right)]}{\sum_{\boldsymbol{j\in[1,J]}} \exp[v\left( \boldsymbol{x}_{j};\hat{\boldsymbol{\beta}} \right)]}$ | (1) |
| --- | --- |

Where $P_{j}$ is the probability of treatment profile $j$ being on average preferred over all available alternatives, $\boldsymbol{x}_{j}$ is the vector of attributes, and $\hat{\boldsymbol{\beta}}$ are the mean estimates. 95% CI were obtained by taking the 2.5% and 97.5% percentiles of the predicted choice probability distributions.

Treatment profiles used for comparison (shown in Table S2) were based on a meta-analysis of clinical trials comparing the efficacy and safety of direct oral anticoagulants (DOACs) for stroke prevention in patients with atrial fibrillation (Fernandez et al., 2015), including data from the ROCKET-AF and ENGAGE AF-TIMI 48 trials, after adjusting for differences in patients’ baseline stroke risk and the length of follow-up during trials. Fatality rates were obtained from Tervonen et al. (Tervonen et al., 2017), taking into account the uncertainty of the baseline risks. The impact of a fatality rate change or disabling event rate change on the likelihood of one specified DOAC treatment being selected over another was estimated based on a change of -70% to 70%.

### 1.5 Data quality

Three non-experimental choice tasks were included in the DCE but were excluded from analyses:

A repeat-choice task (task 14, repeating the randomly chosen third experimental choice task) tested the stability of participant preferences, with selection of the same treatment option in both initial and repeat tasks indicating stability.

A dominance test presented participants with two treatment options, one of which was designed to be superior in all attribute levels; this task evaluated rationality in choice behavior, indicated when the superior treatment was chosen.

A serial non-participation test identified participants who did not provide accurate responses (e.g., those providing uniformly positioned answers within tasks); participants selecting the same option in all 12 tasks were considered serial non-responders. Response time was also monitored.

### 1.6 Health literacy and numeracy

Health literacy was assessed using a set of brief screening questions (SBSQ). Three items were rated on a five-point scale from 0 (highest problems with reading) to 4 (no problems with reading). The total health literacy score was calculated by averaging the sum of the scores across all three items. Lower total scores reflect greater problems with reading.

Numeracy was assessed via the Numeracy Scale (Lipkus et al., 2001), which consists of five questions, each admitting one correct answer. Participants were given one point for each correctly answered question. The maximum attainable score was thus 5. Lower scores reflect greater problems with numbers.

## Supplementary Figures & Tables

#### **Supplementary Fig. A.1.** Patient recruitment and disposition for the discrete choice experiment completed in 2023 by adults with atrial fibrillation in China, Taiwan, and South Korea (N=307).

Responded

(N=1,039)

Not responded (n=2,261)

Eligible

(N=394)

Excluded (n=645)

- Drop-out or not willing (n=4)
- Did not have atrial fibrillation (n=606)^*^
- Did not agree to provide verbal and online informed consent (n=9)
- Not a resident of China, Taiwan, or South Korea or not able to read, speak, and understand the language (n=4)
- Other reasons (n=22)
- Did not consent to collection of personal data for research use (n=8)
- Duplicate participant (n=10)
- Quota already reached (n=3)^†^

Completed

(N=307)

Did not complete (n=87)

- Drop-out (n=10)
- Failed quality checks (n=47)
- Quota already met (n=30)

Contacted

(N=3,300)

^*^Patients were required to be adults (≥18 years) with a diagnosis of atrial fibrillation who were living in China, Taiwan, or South Korea. Patients were not enrolled if they had a cognitive impairment, acute psychopathology, or insufficient knowledge of the language. ^†^Target recruitment was set to 300 eligible participants.

#### **Supplementary Fig. A.2.** Marginal utilities derived using preference data from adults with atrial fibrillation in China, Taiwan, and South Korea (N=307).


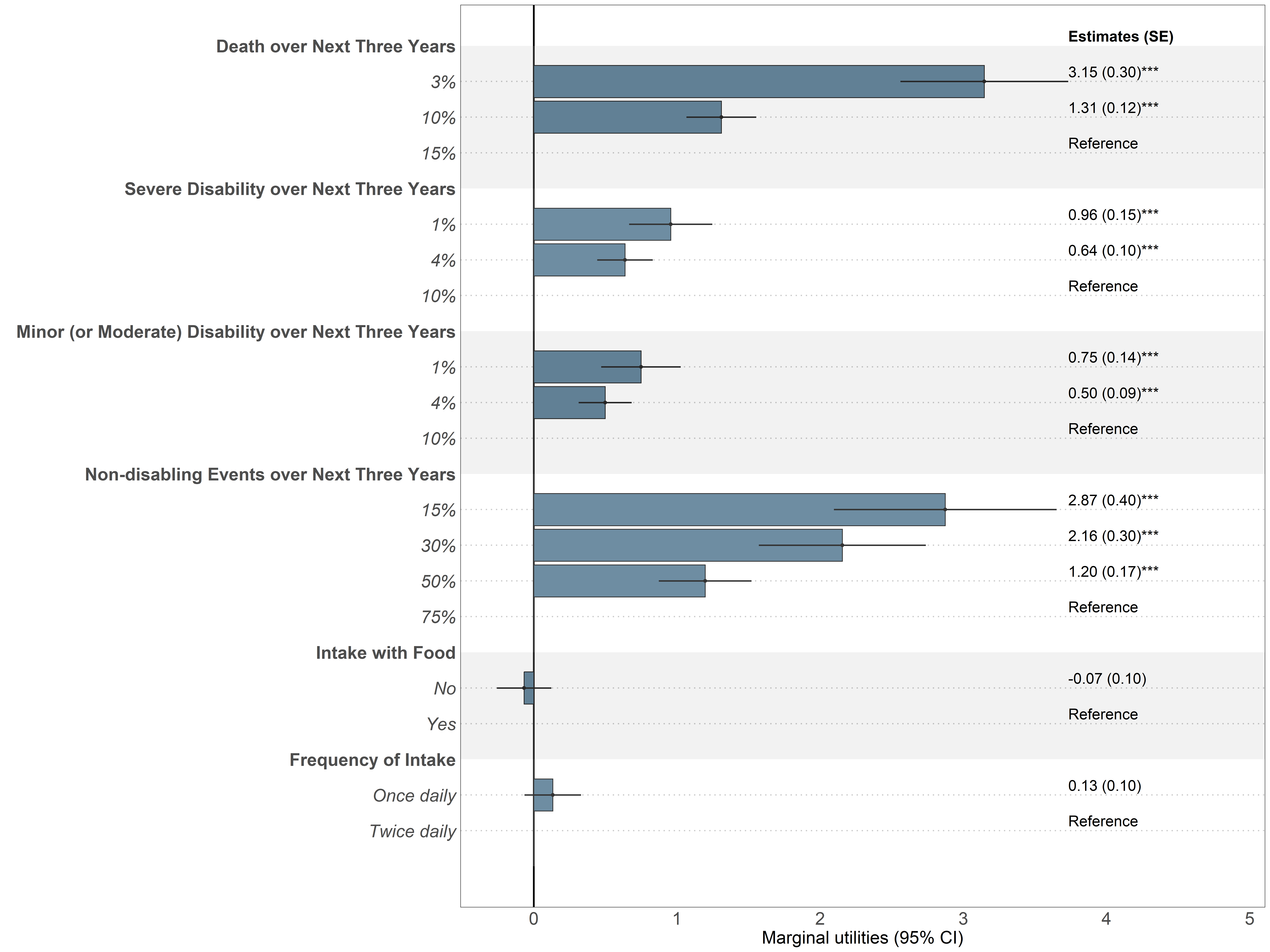


The mean marginal utility and 95% CI of each attribute level, relative to the reference levels included in the DCE, are shown. Estimates are marginal utilities that denote the effect of deviating from a reference level of an attribute on preferences. Positive mean estimates with a 95% CI >0 suggest participants placed a higher value on the attribute level over the reference level which indicates participants preferred the option with the attribute level, all else being equal. *** indicates p<0.001. Abbreviations: CI, confidence interval; DCE, discrete-choice experiment; MLE, maximum likelihood estimates; SE, standard error.

#### **Supplementary Fig. A.3.** Preference estimates for non-vitamin K antagonist oral anticoagulant therapy in adults with atrial fibrillation in China, Taiwan, and South Korea (N=307) by age.


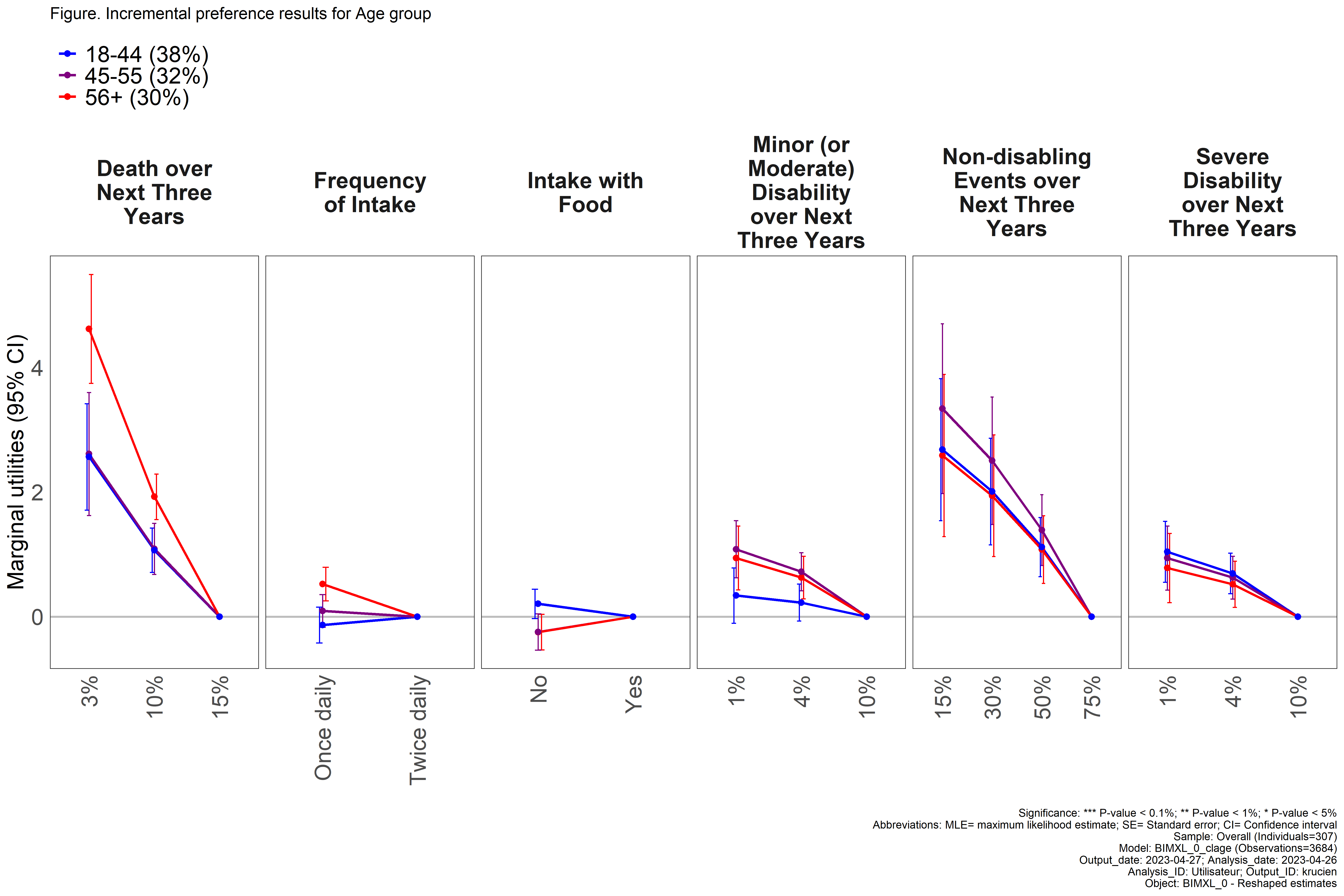


A subgroup analysis was conducted to investigate patient treatment preference heterogeneities by age. The age subgroups were determined based on the retirement age in China (55 years on average for women) and to ensure a sufficient sample size for each subgroup. Interaction effects between the attributes and participant characteristics were used. Participants over 55 years of age placed a significantly higher value (p <0.05) on the risk reduction of death over the next three years, compared to younger patients. Abbreviations: CI, confidence interval.

#### **Supplementary Fig. A.4.** Preference estimates for non-vitamin K antagonist oral anticoagulant therapy in adults with atrial fibrillation in China, Taiwan, and South Korea (N=307) by comorbidities.


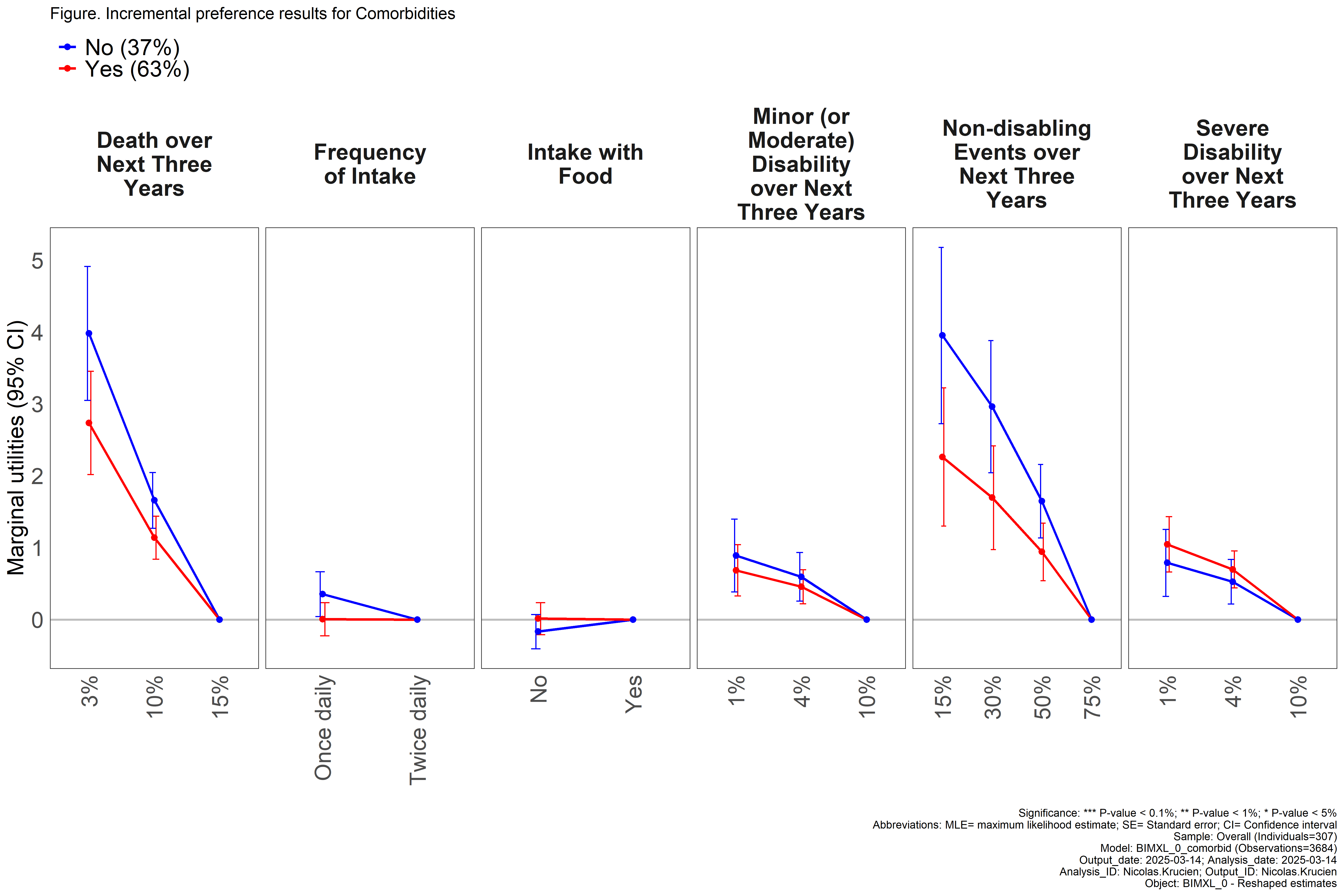


A subgroup analysis was conducted to investigate patient treatment preference heterogeneities by whether or not participants had or had had comorbidities. Interaction effects between the attributes and participant characteristics were used. Participants with comorbidities placed significantly lower values (p <0.05) on the risk reductions of death and non-disabling events over the next three years, compared to those without comorbidities. Abbreviations: CI, confidence interval.

#### **Supplementary Table A.1.** Attributes and levels included in the discrete choice experiment completed in 2023 by adults with atrial fibrillation in China, Taiwan, and South Korea (N=307).

| Attribute | Definition | Levels |
| --- | --- | --- |
| Severe disability over next 3 years | The proportion of patients who will experience severe disability over the next 3 years as a result of an atrial fibrillation-related major stroke or a heart attack, or major bleeding due to a treatment side effect (such as brain bleeding). This will cause permanent damages to health and affect your mobility and daily activities significantly, such as:   - - Your arm and leg are totally paralyzed, and you cannot walk at all.   - Your speech is slurred, and some people cannot understand what you are saying.   - You write and feed yourself with difficulty.   - You spend all of your time either in bed or in a wheelchair.   - You cannot bathe or get dressed without help.   - You need a lot of help at home. If there is not someone at home to care for you, you need to go to a nursing home. | 1% |
|  |  | 4% |
|  |  | 10% |
|  |  | No treatment: 12% |
| Minor (or moderate) disability over next 3 years | The proportion of patients who will experience mild or moderate disability event over the next 3 years as a result of an atrial fibrillation-related moderate stroke or a heart attack, or major bleeding due to treatment side effect (such as the stomach or intestines bleeding). This will cause permanent damages to health and affect your mobility and daily activities, such as:   - - Sometimes you feel shortness of breath, fatigue, and swelling in the ankles and feet.   - Your arm and leg are weak and are hard to move and you will need a walker to walk.   - Your speech is slurred, but people can understand you with occasional difficulty. You have some difficulty writing.   - You need to have some help at home. | 1% |
|  |  | 4% |
|  |  | 10% |
|  |  | No treatment: 12% |
| Death over next 3 years | The proportion of patients who will die as a result of atrial fibrillation-related stroke, heart attack, or some severe treatment side effect. | 3% |
|  |  | 10% |
|  |  | 15% |
|  |  | No treatment: 18% |
| Non-disabling events over next 3 years | The proportion of patients who will experience non-disabling events over the next 3 years, as a result of an atrial fibrillation-related mild stroke or a heart attack, or bleeding (such as nose bleeding) as a treatment side effect. Normally non-disabling events can be resolved after days of rest. | 15% |
|  |  | 30% |
|  |  | 50% |
|  |  | 75% |
|  |  | No treatment: 41% |
| Intake with food | Some medicines have to be taken with a meal, and some medicines do not have this requirement and can be taken independently from food. | Yes |
|  |  | No |
|  |  | No treatment: not applicable |
| Frequency of intake | Some medicines can be taken once a day, and some have to be taken twice a day. | Once daily |
|  |  | Twice daily |
|  |  | No treatment: not applicable |

#### **Supplementary Table A.2.** Health literacy and numeracy scores for the adults with atrial fibrillation in China, Taiwan, and South Korea (N=307) who completed the discrete choice experiment.

| **Characteristics** | **Overall (N=307)** | **China**  **(N=155; 50%)** | **Taiwan (N=76; 25%)** | **South Korea (N=76; 25%)** |
| --- | --- | --- | --- | --- |
| **Health literacy, n (%)** |  |  |  |  |
| Low (≤2) | 75 (24) | 39 (25) | 20 (26) | 16 (21) |
| High (>2) | 232 (76) | 116 (75) | 56 (74) | 60 (79) |
| **How often do you have someone help you read hospital materials? n (%)** |  |  |  |  |
| Always | 8 (3) | 6 (4) | 1 (1) | 1 (1) |
| Often | 51 (17) | 27 (17) | 13 (17) | 11 (14) |
| Sometimes | 73 (24) | 29 (19) | 26 (34) | 18 (24) |
| Occasionally | 94 (31) | 44 (28) | 18 (24) | 32 (42) |
| Never | 81 (26) | 49 (32) | 18 (24) | 14 (18) |
| **How confident are you filling out medical forms by yourself? n (%)** |  |  |  |  |
| Not at all | 0 (0) | 0 (0) | 0 (0) | 0 (0) |
| A little bit | 26 (8) | 15 (10) | 5 (7) | 6 (8) |
| Somewhat | 69 (22) | 35 (23) | 15 (20) | 19 (25) |
| Quite a bit | 111 (36) | 52 (34) | 32 (42) | 27 (36) |
| Extremely | 101 (33) | 53 (34) | 24 (32) | 24 (32) |
| **How often do you have problems learning about your medical condition because of difficulty understanding written information? n (%)** |  |  |  |  |
| Always | 7 (2) | 5 (3) | 2 (3) | 0 (0) |
| Often | 42 (14) | 18 (12) | 16 (21) | 8 (11) |
| Sometimes | 72 (23) | 32 (21) | 21 (28) | 19 (25) |
| Occasionally | 110 (36) | 48 (31) | 25 (33) | 37 (49) |
| Never | 76 (25) | 52 (34) | 12 (16) | 12 (16) |
| **Numeracy skills** |  |  |  |  |
| Low (<3 correct) | 1 (0) | 1 (1) | 0 (0) | 0 (0) |
| High (≥3 correct) | 306 (100) | 154 (99) | 76 (100) | 76 (100) |
| **Which of the following numbers represents the biggest probability/likelihood of getting a disease?** |  |  |  |  |
| Correct: 1 in 10 | 216 (70) | 109 (70) | 48 (63) | 59 (78) |
| Incorrect: 1 in 100 and 1 in 1,000 | 91 (30) | 46 (30) | 28 (37) | 17 (22) |
| **Which of the following represents the biggest probability/likelihood of getting a disease?** |  |  |  |  |
| Correct: 10% | 268 (87) | 134 (86) | 63 (83) | 71 (93) |
| Incorrect: 1% and 5% | 39 (13) | 21 (14) | 13 (17) | 5 (7) |
| **If the chance of getting a disease is 10%, how many people would be expected to get the disease out of 100?** |  |  |  |  |
| Correct: 10 | 304 (99) | 155 (100) | 74 (97) | 75 (99) |
| Incorrect: any numbers other than 10 | 3 (1) | 0 (0) | 2 (3) | 1 (1) |
| **If the chance of getting a disease is 10%, how many people would be expected to get the disease out of 1000?** |  |  |  |  |
| Correct: 100 | 274 (89) | 130 (84) | 71 (93) | 73 (96) |
| Incorrect: any numbers other than 100 | 33 (11) | 25 (16) | 5 (7) | 3 (4) |
| **If the chance of getting a disease is 20 out of 100, this would be the same as having a % chance of getting the disease.** |  |  |  |  |
| Correct: 20 | 298 (97) | 149 (96) | 75 (99) | 74 (97) |
| Incorrect: any numbers other than 20 | 9 (3) | 6 (4) | 1 (1) | 2 (3) |

## Supplementary References

Boxall, P., Adamowicz, W., 2002. Understanding heterogeneous preferences in random utility models: a latent class approach. Environmental & Resource Economics 23:421-446.

Fernandez, M.M., Wang, J., Ye, X., Kwong, W.J., Sherif, B., Hogue, S., Sherrill, B., 2015. Systematic review and network meta-analysis of the relative efficacy and safety of edoxaban versus other nonvitamin K antagonist oral anticoagulants among patients with nonvalvular atrial fibrillation and CHADS2 score ≥2. SAGE Open Med. 3:2050312115613350.

Greene, W.H., Hensher, D.A., 2003. A latent class model for discrete choice analysis: contrasts with mixed logit. Transportation Research Part B: Methodological 37:681-698.

Lipkus, I.M., Samsa, G., Rimer, B.K., 2001. General performance on a numeracy scale among highly educated samples. Med. Decis. Making 21:37-44.

McFadden, D., Train, K., 2000. Mixed MNL models for discrete response. J. Appl. Econ. 15:447-470.

Revelt, D., Train, K., 1998. Mixed logit with repeated choices: households' choices of appliance efficiency level. The Review of Economics and Statistics 80:647-657.

Ryan, M., Watson, V., Entwistle, V., 2009. Rationalising the 'irrational': a think aloud study of discrete choice experiment responses. Health Econ. 18:321-336.

Swait, J., 1994. A structural equation model of latent segmentation and product choice for cross-sectional revealed preference choice data. Journal of Retailing and Consumer Services 1:77-89.

Tervonen, T., Ustyugova, A., Sri Bhashyam, S., Lip, G.Y.H., Verdecchia, P., Kwan, R., Gropper, S., Heinrich-Nols, J., Marsh, K., 2017. Comparison of oral anticoagulants for stroke prevention in nonvalvular atrial fibrillation: a multicriteria decision analysis. Value Health 20:1394-1402.
